# Supplementary material for: Deep learning-based image analysis predicts PD-L1 status from H&E-stained histopathology images in breast cancer
Source: Nat Commun. 2022 Nov 8;13:6753. doi: 10.1038/s41467-022-34275-9 (PMC9643479; doi:10.1038/s41467-022-34275-9)
Supplement: Supplementary file 1 — Supplementary Information [file 41467_2022_34275_MOESM1_ESM.pdf]

Supplementary Figure 1: Visualization of the convolutional neural network architecture.

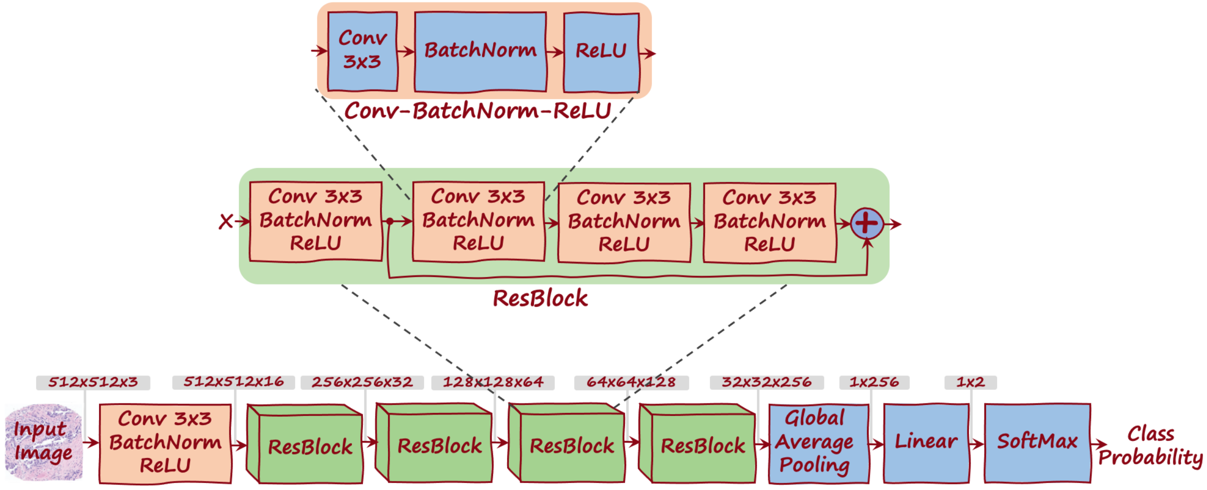

The basic building block of the model is a standard convolution block, consisting of a 3x3 convolution layer followed by a batch normalization layer and a ReLU activation (top). The main backbone (bottom) consists of four main blocks with residual connections that encodes the input to an embedding vector of dimension 256 using a global average pooling layer. The embedding vector is classified to a specific label using a linear layer, followed by a SoftMax activation.

Supplementary Figure 2: Precision-recall and negative predictive value-specificity curves.

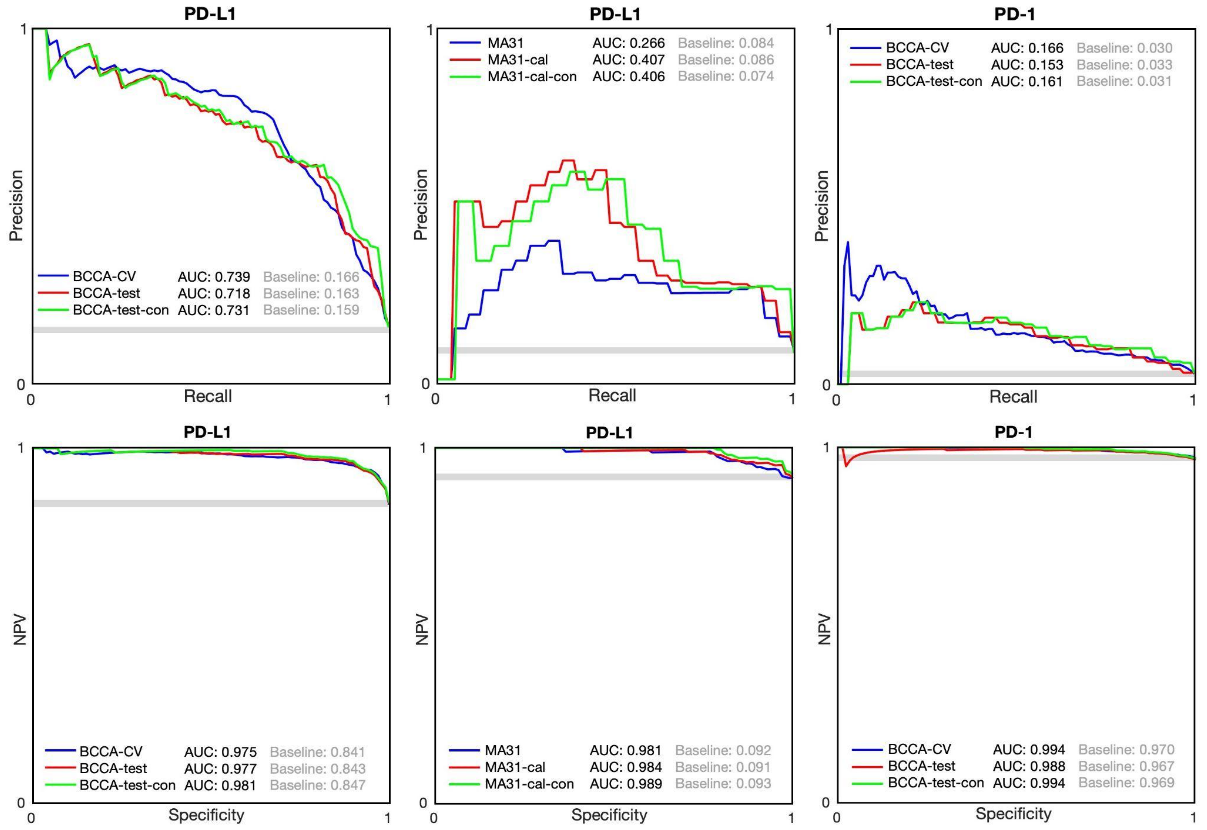

Precision with respect to recall curves and negative predictive value (NPV) with respect to specificity curves, showing the performance of the proposed models for PD-L1 and PD-1 prediction in the BCCA and MA31 cohorts. For each plot, the area under curve (AUC) is noted and compared to the baseline (area under gray line). The baseline states the positive class prevalence in the precision-recall curves, and negative class prevalence in the NPV-specificity curves.

Supplementary Figure 3: t-SNE embedding of the IHC stains.

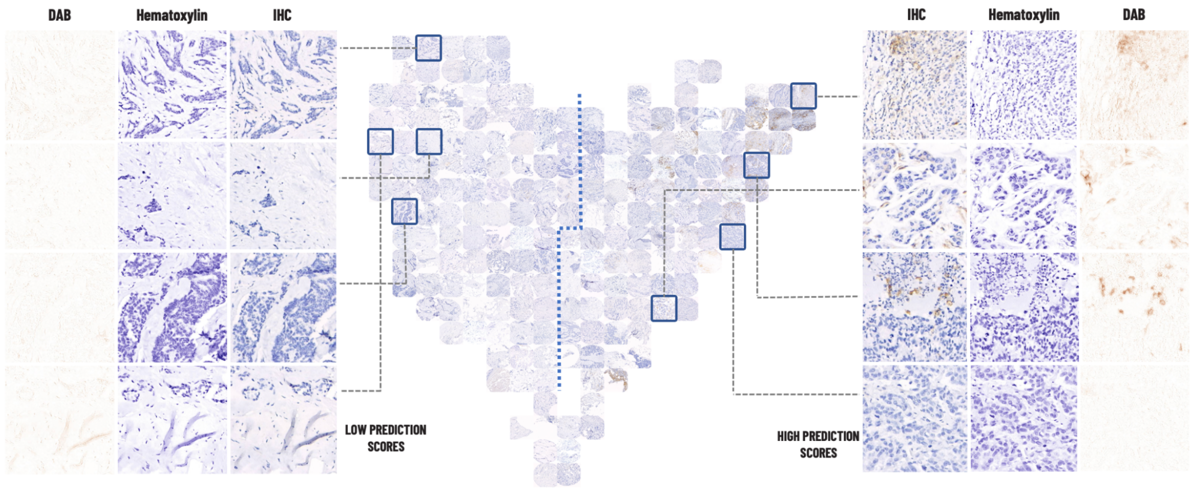

The IHC-stained TMA images corresponding to the t-SNE embedding are presented. Several examples of low and high prediction score images are shown. Each of the IHC examples was deconvolved to DAB and hematoxylin channels, for better visualization.

Supplementary Figure 4: t-SNE and AUC analysis by subtype group.

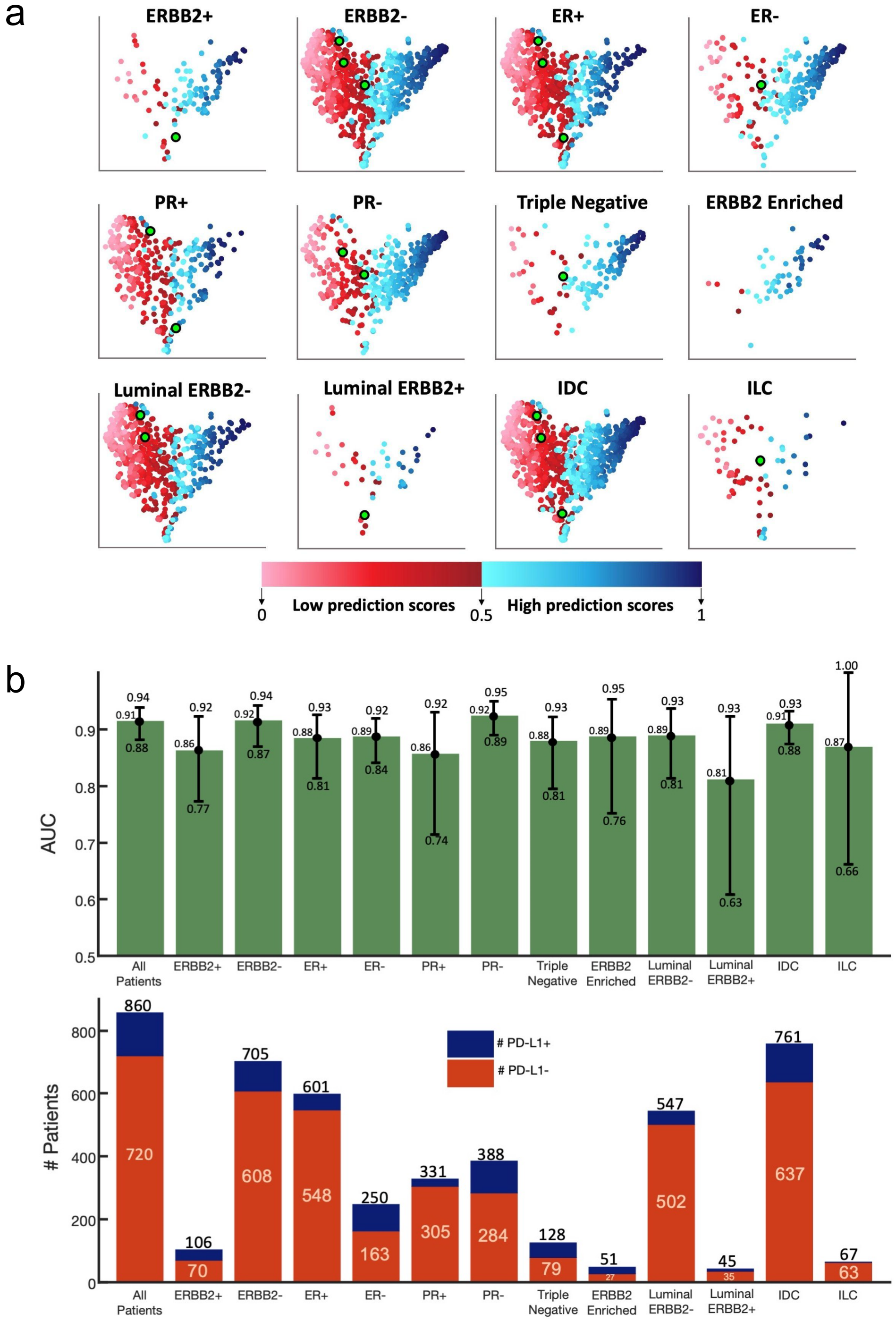

a. The t-SNE distribution for PD-L1 prediction was reproduced for each of the subtype classes in the BCCA test set by keeping only the points corresponding to the patients in the class. The cases that were discordant between the pathologists are marked, showing that they were spread among the subtypes. **b.** The AUC and its 95% CI were computed for each of the classes. The center of each bar represents the AUC of the class, and the error bars show the CIs lower and upper limits. The CIs were computed using bootstrapping, where the number of cases in each class is noted on top of the bars at the bottom. The numbers of positive and total PD-L1 patients per class are presented as well. IDC stands for invasive ductal carcinoma, ILC stands for invasive lobular carcinoma, Triple Negative subtype is defined as ER- PR- ERBB2-, ERBB2 Enriched is defined as ER- PR- ERBB2+, Luminal ERBB2- is defined as (ER+ or PR+) and ERBB2-, and Luminal ERBB2+ is defined as (ER+ or PR+) and ERBB2+.

Supplementary Figure 5: H&E images of discordant versus non-discordant cases in the MA31 cohort.

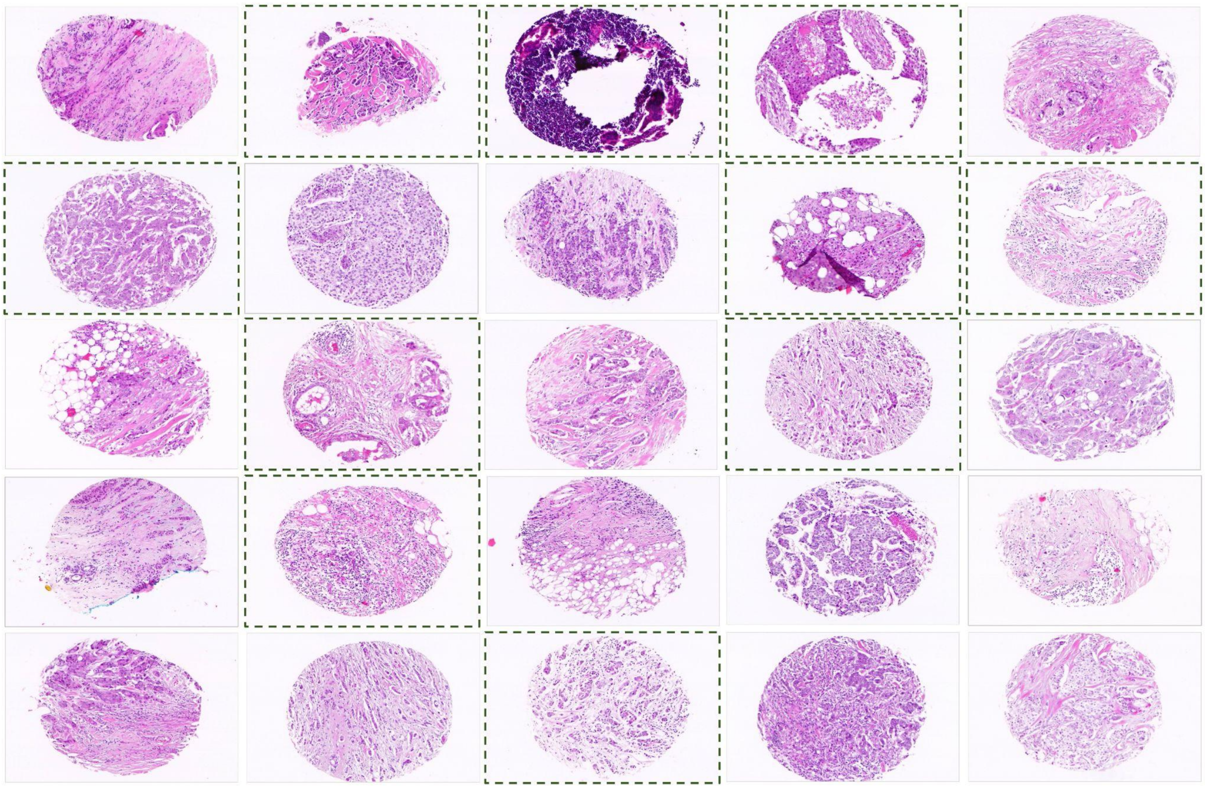

The H&E images of the 10 discordant cases from the MA31 cohort are marked with dashed dark frames. The rest are randomly selected H&E images of the non-discordant cases from the MA31 cohort. The pathologists reported no evident differences in the image features or tissue architecture between the discordant and non-discordant groups.

The Grad-CAM method for better understanding the system's decision making

Grad-CAM [1] is an approach for highlighting image regions, which the AI system mostly relied on for making its prediction, as an attempt to gain a better understanding of the system's decision making. Grad-CAM, and other similar approaches, are commonly used in the context of digital pathology to highlight informative H&E image regions [2,3]. The highlighted regions, however, do not always provide better understanding of the image features themselves used by the system or how they were used to make the prediction. To explore if such an approach would provide a better understanding of how the proposed system predicted the PD-L1 status, we applied the Grad-CAM method and created heat-maps corresponding to the H&E images (Supplementary Figure 6).

The heat-maps highlighted regions in the H&E images that contributed to PD-L1 negative/positive predictions. Nevertheless, it is unclear if this visualization is valuable for better understanding of the features or of the system's decision. The Grad-CAM heat-maps were too coarse for the size of the cells and TMAs in our data. Also, for PD-L1 positive cases, one can expect the highlighted areas to be around the tumor cells expressing PD-L1. However, the highlighted areas were sometimes vague and around areas with no cells, possibly due to our system mostly focused on prediction of the negative class, rather than the positive one.

Supplementary Figure 6: Grad-CAM heat-maps for highlighting informative regions.

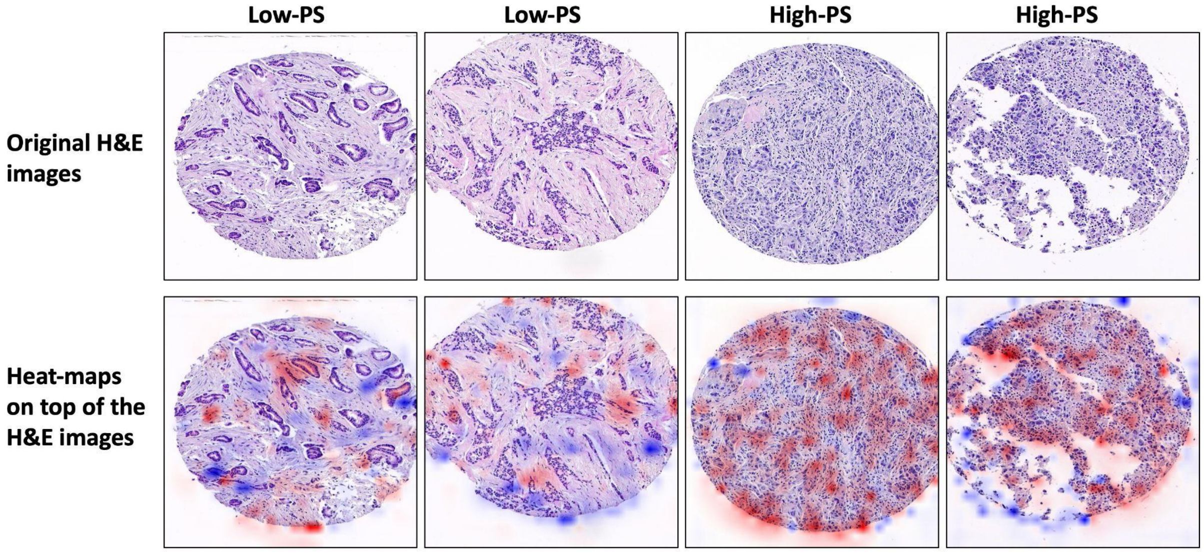

Representative examples of H&E images from the BCCA-test cohort, and their corresponding Grad-CAM heat-maps on top of them. The red and blue colors of the heat-maps highlight regions in the H&E images that contribute to PD-L1 positive and negative prediction, respectively.

Supplementary Table 1: Univariate and Multivariate Analysis.

a. Univariate analysis.

#	Variable	Estimate	Std Error	Wald $\chi 2$	*P* Value	AUC
1	AI score	14.76	1.42	108.31	**< 0.001**	0.915
2	FOXP3-TTIL	2.51	0.82	9.31	**0.002**	0.728
3	CD8-TTIL	2.24	0.81	7.57	**0.006**	0.727
4	Ki-67	2.14	0.83	6.59	**0.010**	0.704
5	EGFR	1.78	0.81	4.87	**0.027**	0.605
6	PR	-1.67	0.79	4.46	**0.035**	0.672
7	ER	-1.64	0.81	4.07	**0.044**	0.695
8	P53	1.36	0.82	2.71	0.100	0.621
9	CK56	1.25	0.79	2.48	0.115	0.535
10	GATA3	-1.22	0.78	2.47	0.116	0.622
11	MDM2	1.17	0.78	2.23	0.135	0.517
12	P-CAHEDRIN	1.18	0.81	2.12	0.145	0.654
13	IGF-1R	-0.92	0.78	1.38	0.239	0.567
14	ERBB2	0.88	0.79	1.23	0.268	0.581
15	CA9	0.83	0.78	1.13	0.287	0.521
16	RET	0.80	0.79	1.03	0.311	0.620
17	CRYAB4000	0.71	0.78	0.84	0.360	0.541
18	HER3	0.60	0.78	0.60	0.439	0.520
19	HER4	-0.19	0.78	0.06	0.804	0.497
20	C-KIT	0.09	0.78	0.01	0.907	0.507

b. Multivariate analysis.

#	Variable	Estimate	Std Error	Wald $\chi2$	*P* Value
1	AI score	10.30	1.08	90.66	**< 0.001**
2	FOXP3-TTIL	1.07	0.27	15.32	**< 0.001**
3	CD8-TTIL	0.87	0.25	11.79	**0.001**
4	Ki-67	0.46	0.26	3.17	0.075
5	GATA3	-0.36	0.26	1.88	0.171
6	P53	0.31	0.27	1.27	0.260
7	MDM2	0.42	0.44	0.92	0.336
8	P-CAHEDRIN	0.23	0.25	0.89	0.346
9	PR	-0.22	0.26	0.75	0.385
10	EGFR	0.26	0.38	0.47	0.495
11	C-KIT	-0.15	0.25	0.34	0.557
12	HER3	0.09	0.39	0.05	0.816
13	ER	0.00	0.29	0.00	1.000
14	ERBB2	0.00	0.31	0.00	1.000
15	CK56	0.00	0.47	0.00	1.000
16	CA9	0.00	0.31	0.00	1.000
17	RET	0.00	0.24	0.00	1.000
18	HER4	0.00	0.29	0.00	1.000
19	CRYAB4000	0.00	0.39	0.00	1.000
20	IGF-1R	0.00	0.57	0.00	1.000

Univariate and Multivariate analysis for PD-L1 prediction in the BCCA test cohort. A logistic regression was fitted to the BCCA training set and applied to the test set. TTILS stands for the total (stromal + intratumoral) tumor infiltrating lymphocytes. The 19 biomarkers were obtained from the publicly available data (see “Methods”) and are described in [2]. The Univariate analysis was done by fitting a logistic regression for each variable separately for prediction of the PD-L1 status. The multivariate analysis was done by fitting an L1-regularized logistic regression for all variables together using the BCCA training data. The Wald Chi-Squared Test was used as the statistical test in both univariate and multivariate analyses. $P < 0.05$ with a 1-tailed hypothesis test indicated statistical significance. The regularization was chosen as the one obtaining the optimal fit, and then the model was applied to the BCCA test set. Since we used an L1 regularization that was optimized on the training set, no adjustment was needed for multiple comparisons on the test set.

Supplementary References

1. Selvaraju, Cogswell & Das. (2017) Grad-cam: Visual explanations from deep networks via gradient-based localization. *Proc. Estonian Acad. Sci. Biol. Ecol* (2017).
2. Shamai, G. *et al.* Artificial Intelligence Algorithms to Assess Hormonal Status From Tissue Microarrays in Patients With Breast Cancer. *JAMA Network Open* vol. 2 e197700 Preprint at https://doi.org/10.1001/jamanetworkopen.2019.7700 (2019).
3. Bychkov, D. *et al.* Deep learning identifies morphological features in breast cancer predictive of cancer ERBB2 status and trastuzumab treatment efficacy. *Sci. Rep.* **11**, 4037 (2021).
