# Supplementary material for: Deep learning-based image analysis predicts PD-L1 status from H&E-stained histopathology images in breast cancer
Source: Nat Commun. 2022 Nov 8;13:6753. doi: 10.1038/s41467-022-34275-9 (PMC9643479; doi:10.1038/s41467-022-34275-9)
Supplement: Supplementary file 3 — Reporting Summary [file 41467_2022_34275_MOESM3_ESM.pdf]

nature portfolio

Corresponding author(s): Gil Shamai

Last updated by author(s): Oct 12, 2022

Reporting Summary

Nature Portfolio wishes to improve the reproducibility of the work that we publish. This form provides structure for consistency and transparency in reporting. For further information on Nature Portfolio policies, see our Editorial Policies and the Editorial Policy Checklist.

Statistics

For all statistical analyses, confirm that the following items are present in the figure legend, table legend, main text, or Methods section.

n/a	Confirmed	
☐	☒	The exact sample size (*n*) for each experimental group/condition, given as a discrete number and unit of measurement
☐	☒	A statement on whether measurements were taken from distinct samples or whether the same sample was measured repeatedly
☐	☒	The statistical test(s) used AND whether they are one- or two-sided<br>*Only common tests should be described solely by name; describe more complex techniques in the Methods section.*
☐	☒	A description of all covariates tested
☒	☐	A description of any assumptions or corrections, such as tests of normality and adjustment for multiple comparisons
☐	☒	A full description of the statistical parameters including central tendency (e.g. means) or other basic estimates (e.g. regression coefficient) AND variation (e.g. standard deviation) or associated estimates of uncertainty (e.g. confidence intervals)
☐	☒	For null hypothesis testing, the test statistic (e.g. *F*, *t*, *r*) with confidence intervals, effect sizes, degrees of freedom and *P* value noted<br>*Give P values as exact values whenever suitable.*
☒	☐	For Bayesian analysis, information on the choice of priors and Markov chain Monte Carlo settings
☒	☐	For hierarchical and complex designs, identification of the appropriate level for tests and full reporting of outcomes
☐	☒	Estimates of effect sizes (e.g. Cohen's *d*, Pearson's *r*), indicating how they were calculated

Our web collection on statistics for biologists contains articles on many of the points above.

Software and code

Policy information about availability of computer code

Data collection	The data used in this research is a public dataset.<br>A computer aided application for fast annotation was developed to assist with the annotation of the data, and is available at: https://github.com/amirlivne/PD-L1_Annotator.
Data analysis	Statistical analysis was performed using Matlab R2019a.<br>Data processing was performed using Python (version 3.7). Specifically, Data pre-processing was based on imgaug library (https://github.com/aleju/imgaug). The model and prediction of PD-L1 status was implemented using Pytorch library (version 1.10.2+cu113). The trained model can be accessed and used at https://github.com/amirlivne/PD-L1_predictor.

For manuscripts utilizing custom algorithms or software that are central to the research but not yet described in published literature, software must be made available to editors and reviewers. We strongly encourage code deposition in a community repository (e.g. GitHub). See the Nature Portfolio guidelines for submitting code & software for further information.

Data

Policy information about availability of data

All manuscripts must include a data availability statement. This statement should provide the following information, where applicable:

- Accession codes, unique identifiers, or web links for publicly available datasets
- A description of any restrictions on data availability
- For clinical datasets or third party data, please ensure that the statement adheres to our policy

The database was composed from a publicly available tissue microarray (TMA) library, published by the Genetic Pathology Evaluation Centre. The TMA datasets can be downloaded from http://bliss.gpec.ubc.ca by navigating to 02-008 for the BCCA cohort and to MA31 for the MA31 cohort.

Human research participants

Policy information about studies involving human research participants and Sex and Gender in Research.

Reporting on sex and gender	The study was based on data of 5,596 females with breast cancer from two publicly available cohorts. No patient recruitment was performed as part of this study. The analysis was agnostic to sex/gender so that sex/gender were not considered in the design of the study.
Population characteristics	Digital images of tissue microarrays from patients that were diagnosed with breast cancer. The data was obtained from the Genetic Pathology Evaluation Centre.
Recruitment	No patient recruitment was performed.
Ethics oversight	The Genetic Pathology Evaluation Centre.

Note that full information on the approval of the study protocol must also be provided in the manuscript.

Field-specific reporting

Please select the one below that is the best fit for your research. If you are not sure, read the appropriate sections before making your selection.

☒ Life sciences ☐ Behavioural & social sciences ☐ Ecological, evolutionary & environmental sciences

For a reference copy of the document with all sections, see nature.com/documents/nr-reporting-summary-flat.pdf

Life sciences study design

All studies must disclose on these points even when the disclosure is negative.

Sample size	The study was based on data of 5,596 patients with 26,763 TMA images from two publicly available cohorts: BCCA (4,944 patients) and MA31 (652 patients). We used all available data for the study to construct and test the system.
Data exclusions	1,945 patients were excluded from the study due to one of the following predetermined reasons: Missing TMA images, no tissue, no tumor, deficient or non-specific staining, or images out of focus.
Replication	We constructed a system based on a machine learning model that was trained and applied to the data. Applying our trained model on the data reproduces the same outcome we obtained in the study.
Randomization	The BCCA cohort was randomly split to a training set (75%) that was used to construct and validate the system and a test set (25%) that was used to test the system. The MA31 cohort was then used for another validation of the system. Randomization of patients for cross-validation was performed completely at random without any stratification.
Blinding	When performing the annotation, the pathologists were blinded to the patient ID of the images and the outcome of the system. Because no treatments were applied in this study, there was no need for further blinding.

Reporting for specific materials, systems and methods

We require information from authors about some types of materials, experimental systems and methods used in many studies. Here, indicate whether each material, system or method listed is relevant to your study. If you are not sure if a list item applies to your research, read the appropriate section before selecting a response.

Materials & experimental systems

n/a	Involved in the study
☒	☐ Antibodies
☒	☐ Eukaryotic cell lines
☒	☐ Palaeontology and archaeology
☒	☐ Animals and other organisms
☒	☐ Clinical data
☒	☐ Dual use research of concern

Methods

n/a	Involved in the study
☒	☐ ChIP-seq
☒	☐ Flow cytometry
☒	☐ MRI-based neuroimaging
